# Perceived Discomfort and Voluntary Activation of Quadriceps Muscle Assessed with Interpolated Paired or Triple Electrical Stimuli

**DOI:** 10.3390/ijerph20064799

**Published:** 2023-03-09

**Authors:** Petra Prevc, Nina Misotic, Igor Stirn, Katja Tomazin

**Affiliations:** Faculty of Sport, University of Ljubljana, 1000 Ljubljana, Slovenia

**Keywords:** interpolated twitch technique, femoral nerve stimulation, knee extension, variability, reliability

## Abstract

Voluntary drive of the exercising muscle is usually assessed with the interpolated twitch technique (ITT), using paired supramaximal electrical stimuli. The aim of this study was to directly compare voluntary activation (VA) of the quadriceps muscle (QM) measured with the ITT, using paired and triple electrical stimuli during maximal voluntary isometric contraction (MVIC). In addition, perceived discomfort was compared with the use of paired and triple electrical stimuli during ITT. Ten healthy participants (23.6 ± 1.6 years) were included. They performed four MVIC, with paired or triple stimuli, in random order. MVIC torque, superimposed evoked torque, evoked torque at rest, VA, and visual analogue scale for pain (VAS-pain), were analysed. The amplitude of the triplet-evoked torque was higher than doublet-evoked torque, i.e., the signal-to-noise ratio increased. However, the differences between the estimation of VA with paired and triple stimuli were not significant (*p* = 0.136). Triple stimuli yielded higher VAS-pain scores than paired stimuli (*p* = 0.016). The limits of agreement for the VA using the Bland–Altman method were 7.66/0.629. It seems that the use of additional electrical stimuli is not a recommended solution for the evaluation of VA, because the advantages (i.e., better signal-to-noise ratio) do not outweigh the disadvantages (i.e., an increase in pain).

## 1. Introduction

Quantifying voluntary activation (VA) of the quadriceps muscle is important because it is the most important locomotor muscle, and is therefore frequently investigated in exercise studies and clinical situations [1,2,3,4]. In particular, quadriceps weakness is frequently studied in anterior cruciate ligament injuries [5,6,7], because a clear distinction between neural and muscular factors contributing to this weakness is critical for optimizing rehabilitation interventions [8,9]. In recent years, VA of the quadriceps muscle has been commonly measured and quantified using the interpolation twitch technique (ITT), in which an electrical or magnetic pulse is applied transcutaneously to the muscle or femoral nerve during maximal voluntary knee extension [10]. Accordingly, ITT typically compares the amplitude of the double-evoked superimposed torque during the plateau of maximal voluntary isometric torque to the amplitude of the double-evoked torque at rest [8]. In the ITT, the proportion of the control twitch that remains superimposed during a maximal voluntary isometric contraction (MVIC) is considered an indicator of the proportion of muscle that is not activated, and therefore the ratio of the superimposed evoked torque to the evoked torque at rest is commonly referred to as the activation deficit. Voluntary activation is then calculated as follows: [1 − superimposed evoked torque/evoked torque at rest × 100] [10]. However, one of the limitations of the observed percentage of VA is the large variability between repeated measurements [11], which can be explained, at least in part, by the ITT method chosen (e.g., type and number of superimposed stimuli) [12]. In addition, it has been suggested that variability between repeated measurements of VA may be due, not only to methodological inadequacies, but also to perceived discomfort during electrical stimulation. Discomfort induced by electrical stimulation during maximal voluntary contractions has been mentioned in several studies [13,14,15]. In addition, pain associated with noxious stimuli impairs the ability to perform maximal voluntary contraction [16].

In recent years, paired electrical stimuli (interpulse interval 10 ms) have typically been administered during voluntary isometric contraction, with the ITT used to determine the percentage of VA. It has been suggested that the use of multiple electrical stimuli with the ITT improves the signal-to-noise ratio [17] and provides a more sensitive tool for measuring VA with the ITT [18,19]. In measurements of voluntary activation, the signal-to-noise ratio refers to the ratio between the amplitude of the superimposed torque and the amplitude of the fluctuations in voluntary force. As VA increases, the amplitude of the superimposed torque decreases significantly, while the fluctuations in voluntary force increase, resulting in a decrease in the signal-to-noise ratio [20]. Therefore, the sensitivity of the ITT is particularly important for measuring VA near the MVIC, where VA is usually tested. Increasing the number of interpolated electrical stimuli could decrease the variability in the amplitude of the superimposed torque, which could increase the sensitivity of the ITT [12,18]. On the other hand, a higher number of interpolated electrical stimuli leads to greater discomfort during VA measurements, which offers questionable advantages when using more than one or two electrical stimuli [16]. Miller et al. [21] showed that the electrical stimulation caused moderate-to-severe discomfort in some participants during some trials, but that the majority of the trials caused mild-to-moderate discomfort.

Although many studies have used different combinations of the number of stimuli [17,22,23], a direct comparison of ITT with paired and triple electrical stimuli (interpulse interval 10 ms) during MVIC, has never been performed. Therefore, the main objective of this study was to compare the ability of ITT with paired and triple electrical stimuli (interpulse interval 10 ms), to detect voluntary activation of the quadriceps muscle in healthy participants during MVIC. Furthermore, it remains to be tested whether higher reliability and lower variability in superimposed triple evoked torques and VA, are obtained. In addition, the subjective discomfort elicited by the two different electrical stimulations was assessed using the visual analogue scale for pain (VAS-pain). We hypothesised that a higher number of interpolated electrical stimuli would improve the estimation of VA, due to lower variability in superimposed triple evoked torques and a higher signal-to-noise ratio. We also hypothesised that ITT with triple electrical stimuli would improve detection and quantification of VA but would simultaneously increase subjective discomfort.

## 2. Materials and Methods

### 2.1. Participants

Ten male physical education students (23.6 ± 1.6 years; 79.5 ± 6.7 kg; 178.8 ± 5.4 cm) participated in the experiment. All participants were free of musculoskeletal pain or injury. Participants were informed of potential risks and signed an informed consent form before the experiment. The Student Affairs Commission of the University of Ljubljana, Faculty of Sport, approved the study (No. 1798/12) to ensure that all ethical standards were met and that the study was conducted in accordance with the Declaration of Helsinki.

### 2.2. Experimental Procedures

Participants came to the laboratory twice, but were tested only on the second occasion. The experimental protocol consisted of four unilateral voluntary and simulated isometric contractions of the right knee extensors, to determine MVIC quadriceps torque, voluntary activation, and evoked torque at MVIC and at rest. The main outcomes were (1) the MVIC torque, (2) the superimposed doublet-evoked torque (TS_D_), (3) the superimposed triplet-evoked torque (TS_T_), (4) the doublet-evoked torque at rest (TR_D_), (5) the triplet-evoked torque at rest (TR_T_), (6) the voluntary activation assessed with paired electrical stimuli (VA_D_), (7) the voluntary activation assessed with triple electrical stimuli (VA_T_), and (8) VAS-pain.

#### 2.2.1. Participant Positioning and Torque Recordings

All measurements were performed under isometric conditions, with the participant comfortably seated on a chair within the frame of an isometric knee torque extension device (our own design). Our custom-built device for measuring isometric knee torque was equipped with two levers. The two levers were rigidly connected at a fixed angle (60°). The force sensor (MES, Maribor, Slovenia) was perpendicular to the axis of rotation (i.e., knee extension) and was attached to the frame at a fixed (non-adjustable) distance from this axis. The second lever was adjustable to the leg length of the participants. Thus, the lower leg was always attached to the isometric frame in the same position, namely 3 cm above the ankle (i.e., the lateral malleolus). Two lever systems guaranteed the constant moment arm of the force sensor to the moment at the knee (i.e., the moment of a force measured by the sensor). Participants were set in a device for measuring isometric knee torque, with the right knee at an angle of 60° (0° full extension) and an angle between the trunk and thigh of approximately 110°. During voluntary and evoked knee extension, participants’ backs were supported and their hips were firmly fixed. Additionally, they were held at the pelvis and above the chest on the isometric frame, to ensure that the upper body did not contribute to the torque of the knee extensors. Participants were also instructed to hold on to the arm supports on either side of the frame. The rotational axis of the dynamometer was visually aligned with the rotational axis of the knee (i.e., lateral femoral epicondyle). During maximal voluntary isometric knee extension, voluntary and evoked torque were measured with a calibrated strain gauge (MES, Maribor, Slovenia), operating linearly in the range of 0–500 N. Responses are expressed as torque about the knee (Nm). A low-noise amplifier was connected to an analogue-to-digital converter (16/30—ML880/P, ADInstruments, Bella Vista, Australia) and a PC with LabChart software (version 7.3.7, ADInstruments, Bella Vista, Australia), to record the torque of knee extension at 1000 Hz.

#### 2.2.2. Electrical Stimulation

An introductory session was held two to five days before the test was performed, to orient participants and familiarise them with the ITT. The supramaximal current intensity was carefully determined on an individual basis (see below). Transcutaneous electrical stimulation (square-wave pulses of 0.1 ms, 400 V maximum voltage) was performed with a constant-current stimulator (Digitimer DS7AH, Hertfordshire, UK). Electrical stimulation was delivered by the cathode, a self-adhesive electrode (KendallTM, ARBOTM, H124SG, COVIDIEN, Gosport Hampshire, UK, 24 mm), which was pressed into the femoral triangle to stimulate the femoral nerve. The anode, a 10 × 5 cm self-adhesive stimulating electrode (Medicompex SA, Ecublens, Switzerland), was placed in the gluteal fold. After positioning of the electrode and submaximal habituation trials, the current intensity of each stimulus was gradually increased by 50 mA, until no further increase in single twitch torque was observed despite further current increase. The current at maximum twitch torque was increased by a further 20%, to ensure a supramaximal stimulus (range: 250–700 mA). Subsequently, only supramaximal paired and triple stimuli (both with an interpulse interval of 10 ms) were delivered, both during and after MVIC.

#### 2.2.3. Assessment of Subjective Discomfort

Subjective discomfort was measured using the visual analog scale for pain (VAS-pain). The VAS-pain is commonly used to assess differences in pain intensity and the effects of different treatments. Participants were instructed to rate their perceived discomfort from the electrical stimuli during quadriceps activation, after the first trial of paired and after the first trial of triple electrical stimulation, on a VAS-pain instrument with a sliding cursor. On the side of the VAS-pain instrument presented to the participant was a broad, unmarked line, 100 mm long. At the left end of the line was the text “No pain”, and at the right end was the text “Worst pain imaginable”. Participants moved the cursor to estimate their discomfort between these two extremes. After each assessment, the position of the cursor was recorded on a marked 100 mm line on the back. At no time during the assessment of discomfort were participants spoken to. Participants were never informed of their ratings.

#### 2.2.4. Experimental Phase

**Figure 1:**
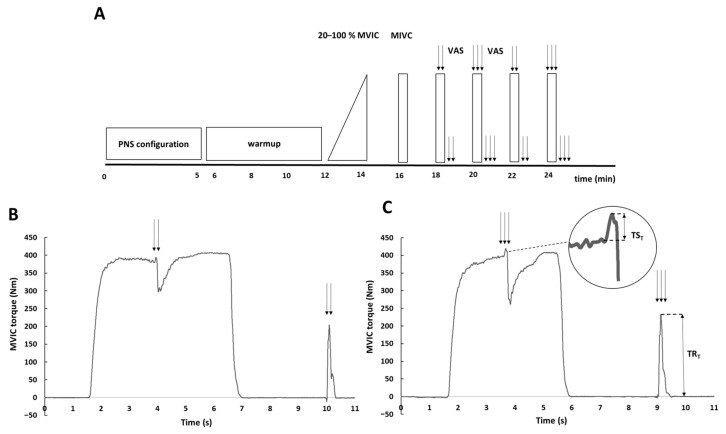
Overview of the neuromuscular testing protocol ((**A**)—timeline of the experiment; (**B**,**C**)—typical examples of raw torque curves over time for one participant). Supramaximal paired (**B**) and triple (**C**) stimuli were interpolated during and immediately after MVIC (maximal voluntary isometric contraction). PNS—peripheral nerve stimulation; ↓↓—paired electrical stimuli; ↓↓↓—triple electrical stimuli; VAS—visual analogue scale; TS_T_—superimposed triplet-evoked torque; TR_T_—resting triplet-evoked torque.

A standardised warm-up exercise consisted of 6 min of stepping on a 20 cm high bench, at a frequency of 0.5 Hz. After stepping, participants performed five isometric knee extensions every 20 s, at 20%, 40%, 60%, 80%, and 100% of the estimated maximum voluntary torque of the knee extensors (Figure 1). Two minutes after warm-up, an MVIC contraction was performed, without regard to the rate of torque development (participants were instructed to “contract as hard as they can”). Subsequently, participants performed four MVIC every two minutes, with paired or triple electrical stimuli interpolated in randomised order across the plateau of voluntary torque. The duration of voluntary contraction was approximately 5 s. Supramaximal paired or triple electrical stimuli were delivered manually 2–3 s after contraction onset, to produce superimposed doublet-evoked torque (TS_D_) or superimposed triplet-evoked torque (TS_T_). Participants were asked to hold the plateau for 1–2 s after superimposed stimulation. Paired or triple electrical stimuli were also delivered at rest, 3 s after each MVIC trial (Figure 1B,C), to produce a doublet-evoked (TR_D_) and a triplet-evoked torque at rest (i.e., as a control) (TR_T_). Visual feedback (instantaneous torque-time curves on a computer screen) and consistent verbal encouragement from the experimenter were always provided.

#### 2.2.5. Data Analysis

MVIC torque was always defined as the highest torque reached before the superimposed stimuli (Figure 1B,C). From the mechanical response elicited by a paired or triple electrical stimuli, the following parameters were determined: (1) superimposed doublet-evoked torque (TS_D_), i.e., the difference between the MVIC torque just before the torque rise and the highest value of the torque curve elicited with paired stimuli, (2) doublet-evoked torque at rest (TR_D_), i.e., the highest value of the torque curve elicited with the paired stimuli at rest (Figure 1B); (3) superimposed triplet-evoked torque (TS_T_), i.e., the difference between the MVIC torque just before the torque rise and the highest value of the torque curve elicited with triple stimuli, (4) triplet-evoked torque at rest (TR_T_), i.e., the highest value of the torque curve elicited during triple electrical stimulation (Figure 1C). To determine the percentage of voluntary activation of the quadriceps muscle by interpolating the paired electrical stimuli, voluntary activation (VA_D_) was estimated using the following formula: (1 − TS_D_/TR_D_) × 100 (Figure 1B). To determine the percentage of voluntary activation of the quadriceps muscle by interpolation of the triple electrical stimuli, voluntary activation (VA_T_) was estimated using the following formula: (1 − TS_T_/TR_T_) × 100 (Figure 1C). To test whether there were differences between voluntary activation measured with paired electrical stimuli (VA_D_) and voluntary activation measured with triple electrical stimuli (VA_T_), the highest values calculated from the two responses were considered for all parameters.

### 2.3. Statistical Analysis

In the design phase of the study, we used SPSS to perform a power analysis. Based on a previous study [15] analysing VA, the results of the power analysis showed that 12 participants needed to be recruited to achieve a power of 0.80. Therefore, we recruited 12 participants, two of whom were unable to complete the experiment because they were very uncomfortable during electrical stimulation, which has been documented in other studies [13,14,24].

Descriptive statistics were calculated for absolute voluntary MVIC torque (i.e., before interpolated electrical stimuli), for superimposed doublet-evoked and triplet-evoked torques (TS_D_ and TS_T_), for doublet- and triplet-evoked torques at rest (TR_D_ and TR_T_), and for VA assessed by interpolated paired and triple electrical stimuli (VA_D_ and VA_T_). Statistical analyses were performed using the Statistical Package for Social Sciences (SPSS version 27, Chicago, IL, USA). Normality of data distribution was tested using the Kolmogorov–Smirnov test. The interclass correlation coefficients (ICC) for the test–retest reliability index were calculated according to Koo and Li [25]. Based on the 95% confidence interval of the ICC estimate, values below 0.5, between 0.5 and 0.75, between 0.75 and 0.9, and above 0.90 were indicative of poor, moderate, good, and excellent reliability, respectively. The variability in the measured outcomes between two repetitions was assessed by the coefficient of variation (CV = SD/mean × 100, where SD is the standard deviation of the mean of the two measurements). To test for differences in non-normal data, the Wilcoxon signed-rank test was used. Differences in normal data were assessed with the paired samples *t*-test. The relationship between the VAS-pain score and other VA measures was tested with Spearman’s rank correlation. The significance level was set at *p* < 0.05. In addition, the Bland–Altman plot, with 95% confidence limits of agreement, was used to test the degree of agreement between percentages of voluntary activation using ITT with paired and triple stimuli [26]. For each participant, the difference between the VA_D_ and VA_T_ stimuli was plotted against the mean of the two VA percentages. Horizontal lines were drawn at the mean difference and at the limits of agreement, which were defined as the mean difference ± 1.96 times the SD of differences.

## 3. Results

The intra-session reliability of MVIC was excellent, and variability was less than 5.5% (Table 1). There were no significant differences in CV for evoked torques when voluntary activation of the quadriceps was assessed by interpolated paired and triple supramaximal electrical stimuli (t = 0.74; *p* = 0.477 for superimposed evoked torques and t = −0.29; *p* = 0.779 for evoked torques at rest, respectively, Table 1). Accordingly, there were no significant differences in CV for the VA assessment by interpolated paired and triple supramaximal electrical stimuli (t = −1.16; *p* = 0.276; Table 1). The torque produced by voluntary isometric contraction of the knee extensors was virtually identical before the superimposed doublet- and triplet-evoked torques (t = −0.89, *p* = 0.395). The interpolating triple electrical stimuli induced higher superimposed triplet-evoked torques (10.4 ± 9.0% MVIC for superimposed triplet-evoked and 6.9 ± 5.4% MVIC for superimposed doublet-evoked torques, t = −2.27; *p* = 0.049; power = 0.527; Table 1). Similarly, significant differences were observed between doublet- and triplet-evoked torques at rest (57.8 ± 11.3% MVIC for triplet-evoked and 49.1 ± 7.3% MVIC for doublet-evoked torques at rest, t = −6.1; *p* < 0.001; power = 0.999; Table 1).

**Figure 2:**
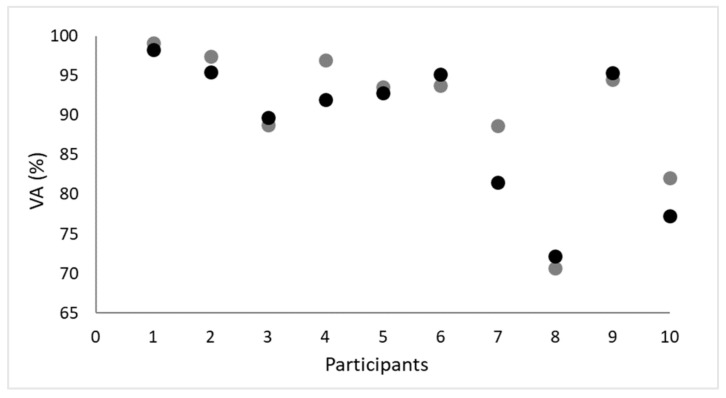
VA (voluntary activation) of the quadriceps muscle for all 10 participants (paired electrical stimuli were used during the ITT, grey circles; triple electrical stimuli were used during the ITT; black circles).

**Figure 3:**
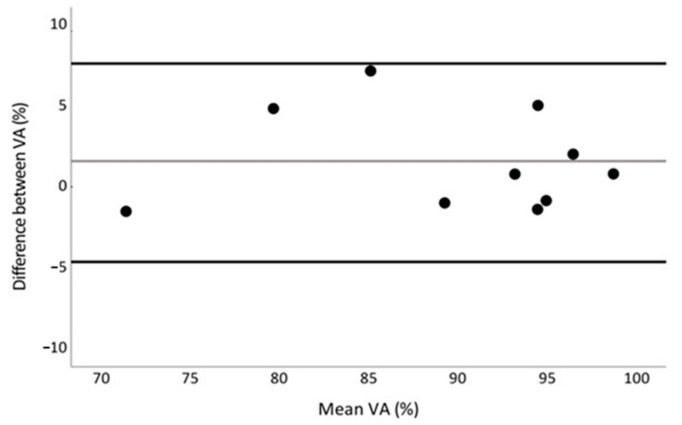
Agreement between the VA assessment by ITT with paired and triple electrical stimuli (Bland–Altman plot). The grey line represents the mean difference (the VA assessed with paired stimuli minus the VA assessed with triple stimuli), and the black lines represent the 95% limits of agreement. Black circles represent value for each participant.

While the amplitude of the triplet-evoked torque was higher than the doublet-evoked torque, i.e., the signal-to-noise ratio increased, the differences between VA_D_ and VA_T_ were not significant (t = 1.6; *p* = 0.136; power = 0.310; Table 1). However, Figure 2 shows that the interpolated paired electrical stimuli overestimated voluntary quadriceps muscle activation by 5.7 ± 1.1% in three participants, whereas a similar percentage of voluntary quadriceps muscle activation was observed in the other participants. The Bland–Altman plot shows that the mean bias ± SD between VA measured with paired and triple stimuli was 1.60 ± 3.09%, and the limits of agreement ranged from −4.46% to 7.66% (Figure 3). On the other hand, participants’ discomfort (VAS-pain) was significantly higher when voluntary quadriceps activation was measured with ITT using triple stimuli (6.1 ± 1.1 mm and 7.7 ± 1.5 mm for paired and triple stimuli, respectively, Z = −2.4, *p* = 0.016; power = 0.798). In addition, participants with higher VAS-pain scores had a higher coefficient of variation for the VA_T_ (Rs = 0.68; *p* = 0.03).

## 4. Discussion

The main objective of this study, was to compare the effects of the number of stimuli on the estimates of voluntary activation of the quadriceps muscle during MVIC in healthy participants. In addition, the subjectively perceived discomfort caused by paired and triple electrical stimuli during ITT was assessed, using the visual analogue scale for pain (VAS-pain). The main findings of this study are that, (1) the amplitude of the evoked torque increased significantly, i.e., the signal-to-noise ratio, when triple versus double electrical stimuli were used during ITT; (2) the variability in the superimposed triplet-evoked torques (TS_T_) remained high and were not significantly different from the superimposed doublet-evoked torques (TS_D_); (3) VA_D_ and VA_T_ showed good reliability (ICC > 0.78); (4) subjectively perceived discomfort increased when an additional electrical stimulus was used during the ITT; (5) participants with higher VAS-pain scores had lower VA_T_ and a higher coefficient of variation for VA_T_.

Mean values for VA_D_ and VA_T_ were ~89% and ~91%, respectively (*p* = 0.136). This is consistent with other studies examining voluntary activation of the fresh quadriceps muscle in healthy participants [20,27,28]. It has been previously shown that even healthy subjects cannot adequately use the total force that their quadriceps muscle can generate, due to insufficient voluntary drive [29]. Furthermore, the intra-session reliability of the ITT with paired and triple electrical stimuli obtained in our study is consistent with previously published results [28]. As expected, our results showed a 1.2-fold increase in TR_T_ compared with TR_D_; furthermore, TS_T_ was approximately 1.5-fold higher compared with TS_D_. Similarly, Allen et al. [23] showed a more than 2-fold increase in the amplitudes of the superimposed torque with paired electrical stimulation compared with a single stimulus. Thus, our study confirms that an additional supramaximal stimulus indeed increases the signal-to-noise ratio. However, for TS_T_, large within-subject variability has been observed, regardless of the additional electrical stimuli used [11,18,27,30]. The large within-subject variability for TS_T_ and TS_D_ could likely be due, not only to the nonlinear relationship between voluntary and superimposed torques, but also to some additional methodological issues [31]. Oskouei et al. [27] showed that a systematic increase or decrease in torque at the time of application of the electrical stimuli, has an important effect on the amplitude of the superimposed torques. For example, when voluntary torque was increasing at the time of stimulus application, the amplitude of the superimposed evoked torque was higher than the superimposed amplitude obtained when voluntary torque remained constant or was decreasing [22,27].

Kooistra et al. [18] claimed that triple stimulation at 300 Hz increases the ITT signal-to-noise ratio. However, in their study, no direct comparison was made with other types of stimulation. Our results show that ITT with an additional electrical stimulus increased triplet-evoked torque compared with doublet-evoked torque (Table 1), whereas the differences between VA_D_ and VA_T_ were not significant (t = 1.6; *p* = 0.136; power = 0.310; Table 1). In addition, the Bland–Altman analysis confirms that ITT with triple stimuli can be used as an alternative to paired stimuli, because there is sufficient agreement between stimulation methods in the assessment of VA. However, in some participants in our study, we observed a discrepancy between VA_D_ and VA_T_ (Figure 2). It appears that in some participants, the paired electrical stimuli, compared to the triple stimuli, were not able to recruit all motor units that are not maximally recruited during voluntary contraction. 

The resting evoked torques elicited with paired and triple electrical stimuli showed low, and not significantly different, variability. Moreover, the reliability of resting evoked torques was excellent for both types of stimuli (ICC > 0.94). It appears that ~5 s MVIC is an efficient conditioning contraction, that produces a constant amplitude of resting torque (i.e., a similar potentiation effect) after the administration of paired or triple electrical stimuli, although TR_T_ was significantly higher than TR_D_ (*p* < 0.001). An additional supramaximal stimulus likely increases the amount of calcium ions released from the sarcoplasmic reticulum into muscle fibers, resulting in an increased number of cross-bridges, which in turn results in a higher triplet-evoked resting torque [32].

Although the Bland–Altman analysis shows sufficient agreement between the ITT with paired and triple stimuli for the assessment of VA, the use of additional electrical stimuli does not seem to be a useful strategy to increase the signal-to-noise ratio during the ITT, because perceived discomfort increased significantly when triple electrical stimuli were administered. Specifically, our results showed that the mean VAS-pain score for interpolated paired electrical stimuli was lower (~61 mm), and classified as moderate, than the mean VAS-pain for interpolated triple electrical stimuli (~77 mm), which was classified as severe. In addition, participants with higher VAS-pain scores had a higher coefficient of variation for the VA_T_ (Rs = 0.68; *p* = 0.03). It has been speculated that an activation deficit, due to the anticipation of a noxious stimulus, could cause greater variability in volitional drive [16], which may also be the case in our study.

There are some limitations to this study. First and foremost, the small sample size of this study is a clear limitation that affects the generalizability of the results obtained. Accordingly, the statistical power for the main results ranges from 31% to 100%. The second limitation of our study is the limited number of repetitions, which could affect the results. However, our participants were very familiar with VA assessment, so they were able to perform consistent MVIC and achieve a clear force plateau. The third limitation of our study may be related to the supramaximal nature of electrical stimulation during ITT. Indeed, the use of multiple stimuli during superimposed stimulation has been shown to give spinal reflexes more time to dissipate the superimposed response [29]. Model calculations suggest that the use of paired stimuli at 100 Hz has a minimal effect on estimates of VA [33]. However, it seems highly unlikely that a higher spinal reflex response was elicited by triple electrical stimuli, as TS_T_ was significantly higher than TD_T_. In addition, the use of a short stimulation pulse, e.g., 0.1 ms, seems unlikely to stimulate afferents that are normally recruited with longer pulses (e.g., 1 ms). In addition, the fourth limitation refers to supramaximal electrical stimulation, which could cause coactivation of the hamstring, because the electrical current also passes through the sciatic nerve. However, we consider it highly unlikely that ITT with triple stimuli would cause coactivation by stimulating the sciatic nerve, because there would be a significant decrease rather than an increase in TR_T_ compared with TR_D_. The fifth constraint refers to the 60° knee flexion angle we used to evaluate voluntary activation (0° full extension). Whereas, most previous studies have used the 90° angle to assess quadriceps strength and activation [9,34,35]. It is unclear whether our results for the 60° knee flexion angle are generalizable to other knee angles, and further research is needed to verify whether our results apply to other knee angles.

However, further studies should compare the behavior of VA_D_ and VA_T_ after acute (e.g., determination of central fatigue and post-activation performance enhancement) and chronic interventions (e.g., strength training).

## 5. Conclusions

In conclusion, quantification of the quadriceps muscle VA using ITT with paired and triple electrical stimuli was not significantly different during MVIC, although the signal-to-noise ratio increased. The use of interpolated triple electrical stimuli increased the discomfort perceived by participants. In addition, when additional electrical stimuli were used, higher VAS-pain scores were associated with higher CV. This study suggests that although ITT with triple supramaximal stimuli allows detection of VA and has acceptable variability, induced discomfort may seriously limit its clinical application in physical therapy or rehabilitation.

## Figures and Tables

**Table 1:** Reproducibility and variability in voluntary and evoked torque measurements during assessment of quadriceps activation with paired and triple supramaximal electrical stimulation.

	Mean [95% CI]	CV	ICC (3,1) [95% CI]
MVIC (Nm)	379.4 ± 83.4 [319.7, 439.1]	5.4 ± 2.4%	0.95 [0.88, 0.99]
TS_D_ (Nm)	22.3 ± 16.0 [10.9, 33.7]	34.5 ± 25.4%	0.90 [0.64, 0.97]
TS_T_ (Nm)	33.7 ± 28.1 [13.6, 53.8]	28.4 ± 14.1%	0.88 [0.56, 0.97]
TR_D_ (Nm)	175.9 ± 30.7 [154.0, 197.9]	2.2 ± 1.7%	0.98 [0.93, 0.99]
TR_T_ (Nm)	211.3 ± 43.2 [180.3, 242.2]	2.5 ± 3.1%	0.95 [0.80, 0.99]
VA_D_ (%)	90.6 ± 8.6 [84.4, 96.8]	3.3 ± 2.9%	0.84 [0.48, 0.96]
VA_T_ (%)	89.0 ± 8.7 [82.6, 95.3]	5.2 ± 4.5%	0.78 [0.34, 0.94]

Legend: CV—coefficient of variation; ICC (3,1)—interclass correlation; MVIC—maximal voluntary isometric contraction torque; TS_D_—superimposed doublet-evoked torque; TS_T_—superimposed triplet-evoked torque; TR_D_—doublet-evoked torque at rest; TR_T_—triplet-evoked torque at rest; VA_D_ (%)—voluntary activation assessed by interpolated paired electrical stimuli; VA_T_ (%)—voluntary activation assessed by interpolated triple electrical stimuli.

## Data Availability

The raw data supporting the conclusions of this article will be made available by the authors, without undue reservation.
